# Contrast enhanced X-ray computed tomography imaging of amyloid plaques in Alzheimer disease rat model on lab based micro CT system

**DOI:** 10.1038/s41598-021-84579-x

**Published:** 2021-03-16

**Authors:** Michaela Kavkova, Tomas Zikmund, Annu Kala, Jakub Salplachta, Stephanie L. Proskauer Pena, Josef Kaiser, Karel Jezek

**Affiliations:** 1 Central European Institute of Technology, Brno University of Technology, Brno, Czech Republic; 2 Faculty of Medicine in Pilsen, Charles University, Pilsen, Czech Republic

**Keywords:** Experimental models of disease, Diagnostic markers, Prognostic markers, Dementia, Neurodegenerative diseases, Alzheimer's disease, Computational biophysics, Neural ageing

## Abstract

Amyloid plaques are small (~ 50 μm), highly-dense aggregates of amyloid beta (Aβ) protein in brain tissue, supposed to play a key role in pathogenesis of Alzheimer’s disease (AD). Plaques´ in vivo detection, spatial distribution and quantitative characterization could be an essential marker in diagnostics and evaluation of AD progress. However, current imaging methods in clinics possess substantial limits in sensitivity towards Aβ plaques to play a considerable role in AD screening. Contrast enhanced X-ray micro computed tomography (micro CT) is an emerging highly sensitive imaging technique capable of high resolution visualization of rodent brain. In this study we show the absorption based contrast enhanced X-ray micro CT imaging is viable method for detection and 3D analysis of Aβ plaques in transgenic rodent models of Alzheimer’s disease. Using iodine contrasted brain tissue isolated from the Tg-F344-AD rat model we show the micro CT imaging is capable of precise imaging of Aβ plaques, making possible to further analyze various aspects of their 3D spatial distribution and other properties.

## Introduction

Alzheimer's disease (AD) is the most common cause of dementia worldwide, extensively interfering with personal, social, and health care levels of human society. AD progression is characterized by a gradual deterioration of cognitive abilities, starting with often unnoticed declarative memory impairment, followed by problems with spatial orientation, and ending with an inability to cope with daily life routine. The rare early onset form can affect individuals in their 40 s or 50 s, but the AD risk rapidly increases after the age of 65. While the etiology of AD remains still unclear, there are several hypothesized mechanisms reflecting molecular changes observed in brain tissue of AD patients, mainly the deposits of amyloid beta and hyperphosphorylated tau protein^1–3^. The classical “amyloid cascade hypothesis”^4^ states that the amyloid beta (Aβ) plaques are the causative agent of Alzheimer's pathology. The Aβ plaques are small (~ 50 μm), dense objects composed of clumped fibrils of amyloid β (Aβ), a peptidic product of the amyloid precursor protein. Their formation induces a local inflammation resulting in progressive cellular loss and related cognitive inability. It has been therefore assumed that the reduction of Aβ deposits might help to control the AD course. Numerous approaches have been designed to reduce the Aβ content in the brain tissue^5^. In order to test the effectiveness of such an AD therapy, there is a substantial need of a non-invasive, precise, and reproducible imaging method to analyze the location and size of the Aβ plaques in the brain.

In the large majority of studies on mouse and rat models, the amyloid plaques were quantified histologically in sliced and stained brain tissue. While the histology and immunohistochemistry still remain important routines for characterizing the plaque properties, imaging techniques represent a powerful tool for a precise 3D analysis of plaque distribution.

So far all the attempts to image the amyloid plaques in rodent brain by Computed Tomography (CT) have been performed on mouse brain (*mus musculus*). The experiments were performed with synchrotron X-ray sources using variations of phase contrast imaging to visualize the Aβ plaques^6–11^. Overall, in all published papers on the topic of Aβ plaques detection, the authors were able to identify the Aβ plaques in the mouse brain using phase contrast imaging, some even performing quantitative analyses^11^. However, imaging at synchrotron facilities is often limited by the narrow field of view resulting in a need of several scans for the imaging of one brain. Also, the access to synchrotron facilities is very limited and burdened by the high price of the CT scans.

Absorption based contrast enhanced X-ray micro CT imaging utilizing a lab based industrial micro CT devices is an emerging imaging technique which uses the staining solutions with high proton number elements to employ the contrast in soft tissues of biological samples which are in their native state invisible to X-rays^12,13^. Contrast enhanced micro CT imaging has been previously proved to be a promising method for a precise 3D analysis of wide variety of samples such as the developing cartilage of nasal capsule of mice^14–17^, development of palate^18,19^, complex tooth shape in reptiles^20^, research of congenital heart and kidneys defects^21^, formation of mammalian neck muscles^22^ and even a noninvasive observation of a human embryo^23^. One of the advantages of micro CT imaging is the possibility to translate a micro CT generated 3D model into a 3D pdf format, which enables an easier communication of obtained data^24^.

The main advantage of absorption based contrast enhanced X-ray micro CT imaging is the possibility to obtain precise 3D information about inner structures of the entire brain without sectioning (which might induce artifacts related to brain deformation or missing tissue due to the sectioning). The micro CT imaging of rodent brain has been previously used for the visualization of vascular system of mouse brain filled with radio-opaque silicone rubber Microfil^25–31^, as a tool for localization of cerebral ischemia^32,33^ to test the efficiency of different micro CT contrasting agents^24–38^, and even for the analysis of specific structures of the brain^39^.

However, it has not been previously determined whether the absorption based contrast enhanced X-ray micro CT imaging is capable of detecting and quantifying amyloid plaques in the brains of rodents. In this study, we optimized the mouse brain staining protocol^40^ for the staining of a much larger rat brain. The lab based industrial micro CT device GE Phoenix v|tome|x L 240 was utilized to scan the contrast enhanced brains of a recent rat AD model to determine whether amyloid plaques could be detected by contrast enhanced micro CT imaging. Further, the exploration of the possibilities of Aβ plaque analyses provided by obtained high resolution micro CT data was pursued. This experimental setup promises a faster, more precise and accessible alternative to the synchrotron based micro CT imaging of the Aβ plaque deposits.

## Results

**Figure 1:**
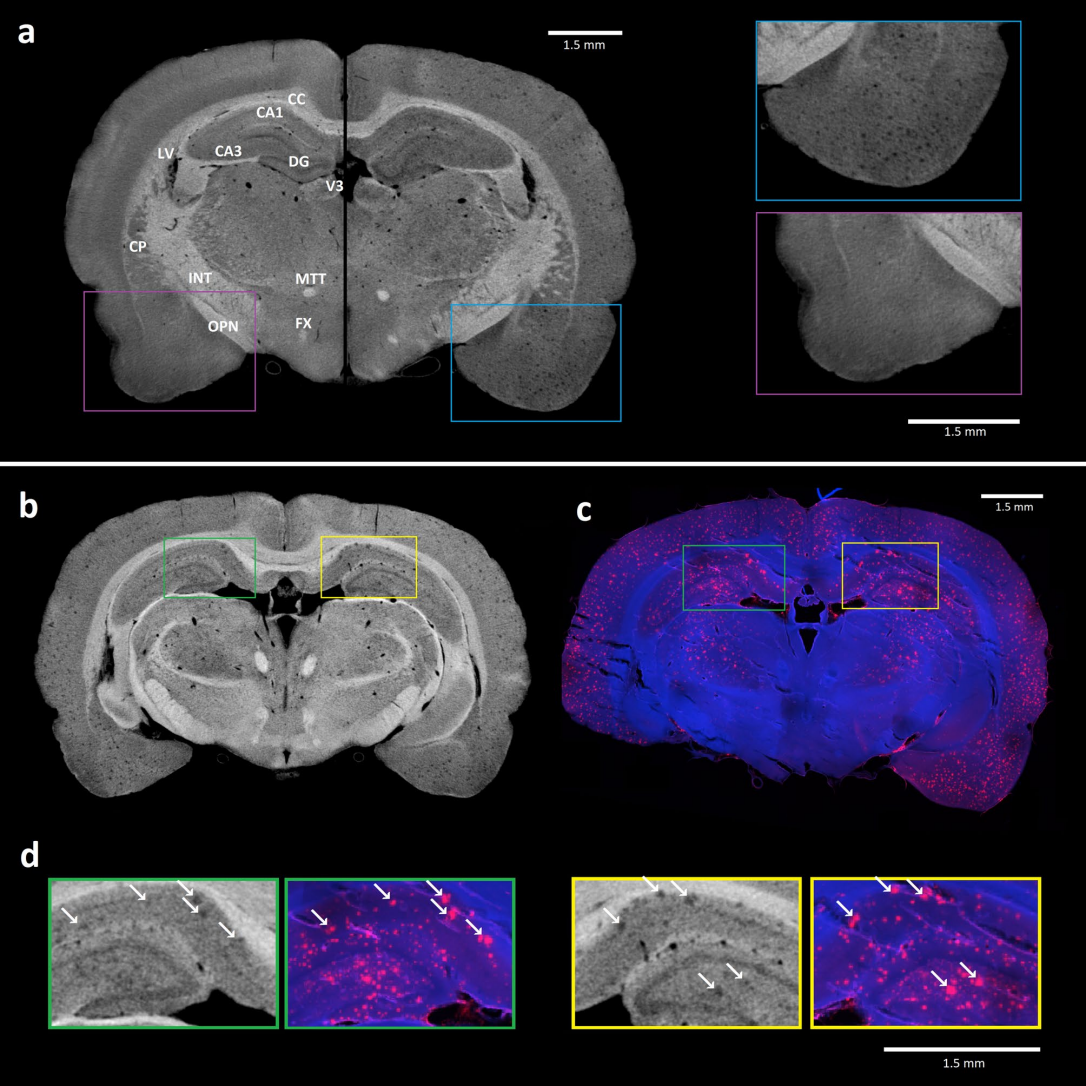
Confirmation of plaques identity in micro CT data: (**a**) Composed picture of the micro CT scan sections from control (left side) and transgenic (right side) rat brain. The transgenic tissue exhibits a large amount of suspect amyloid plaques (right part of the brain). Selected detail image of corresponding areas that are shown in pink and blue frames. Basic anatomical structures visualized in micro CT scan: *CA1 and CA3* cornu Ammonis 1 and 3 of hippocampus, *CC* corpus callosum, *CP* caudoputamen, *DG* dentate gyrus, *FX* fornix, *INT* internal capsule, *LV* lateral ventricle, *MTT* mamillothalamic tract, *OPN* olivary pretectal nucleus, *V3* third ventricle. Micro CT image (**b**) and immunohistology section with detected plaques (**c**) of corresponding coronal brain sections from the transgenic rat no. 60 confirms the presence of plaques in micro CT scan. Selected plaques are indicated by white arrows in enlarged slice windows (**d**)^41^.

We used brains from three 18 months old female rats (two transgenic TgF-344 AD animals and one wild type control). The micro CT scan slices of iodine stained brains from transgenic and wild type samples are shown in the Fig. 1a (for an animated movie see the Supplementary material). Besides the basic anatomical structures visualized equally across both brains, the transgenic brain expressed a large amount of dark grey spots—most likely suspect Aβ plaques that were condensed widely in cortical (neocortex, hippocampus) and some subcortical areas.

Beta amyloid is the main component of amyloid plaques. Because of their dense protein content, we expected the applied iodine based contrasting protocol would preferably stain the tissue around the amyloid plaques, resulting in their “negative” highlight in the micro CT data. The golden standard for beta amyloid detection is its immunostaining by binding to a specific antibody tagged by fluorescent probe. Here, we used this approach to validate the identity of lower density loci detected by micro CT in the transgenic samples. To confirm the identity of the suspect plaques, the iodine stained brain samples were washed in ethanol solution after the micro CT scanning, cut into histological sections and finally specifically stained for the amyloid protein. For selected histological slices we identified their respective sections in the micro CT data. The resulting comparison is displayed in Fig. 1b which shows the corresponding slice as a micro CT image (1b) and as a histological section (Fig. 1c, detailed view in Fig. 1d). The placements of the suspect Aβ plaques overlapped with the immunodetected Aβ plaques positions (arrows in Fig. 1d). This match strongly supports the fact that the introduced micro CT protocol sufficiently detects individual amyloid plaques in ex vivo whole brain.

**Figure 2:**
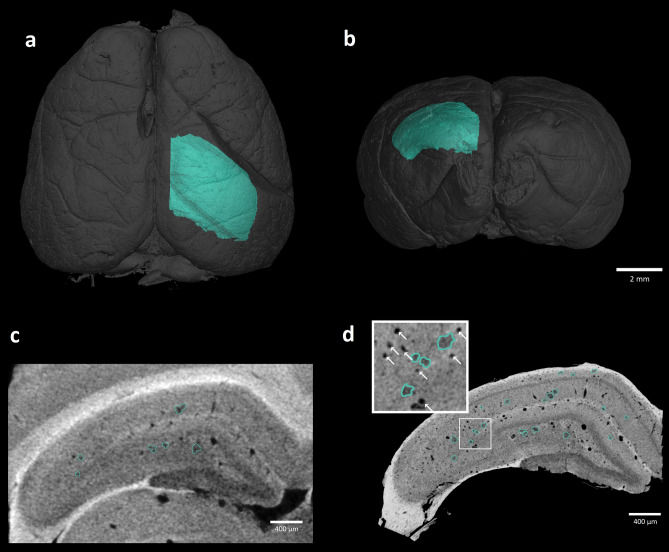
Detailed scan of the dissected dorsal hippocampus: Horizontal (**a**) and coronal (**b**) reconstructed view of the brain with dissected right dorsal hippocampus (green) used for the isolated scan. The same hippocampal tissue sample imaged by two different micro CT approaches (**c**,**d**) where the suspect plaques are highlighted in green. Panel (**c**) shows the section taken from the whole brain scan. Panel (**d**) depicts the same area after the isolated scanning that achieved smaller voxel size, in the magnified image the blood vessels are labeled by white arrows^41,42^.

In the industrial micro CT setups that use the cone beam geometry, the dimension of the sample is one of the main factors that determine the resulting voxel size of the obtained dataset. To acquire a 3D distribution of Aβ plaques in the best possible details, we decided to image an isolated part of the brain and to scan it again with a smaller voxel size. We focused on the dorsal hippocampus as this structure is severely impaired by the amyloid accumulation and cellular loss in AD. The dissected dorsal part of the hippocampus is showed in Fig. 2a,b. While the whole brain scan delivered a voxel size of 9 µm (Fig. 2c), in the case of isolated hippocampus we achieved a voxel dimension of 3 µm (Fig. 2d). The comparison of both scans convincingly shows that the readability of the large amyloid plaques’ borders and especially the visibility of small plaques was compromised, even though they were distinguishable in the whole brain scan. On the contrary, the dissected sample yielded a considerably higher level of detail, enabling to detect a large amount of plaques of various sizes.

**Figure 3:**
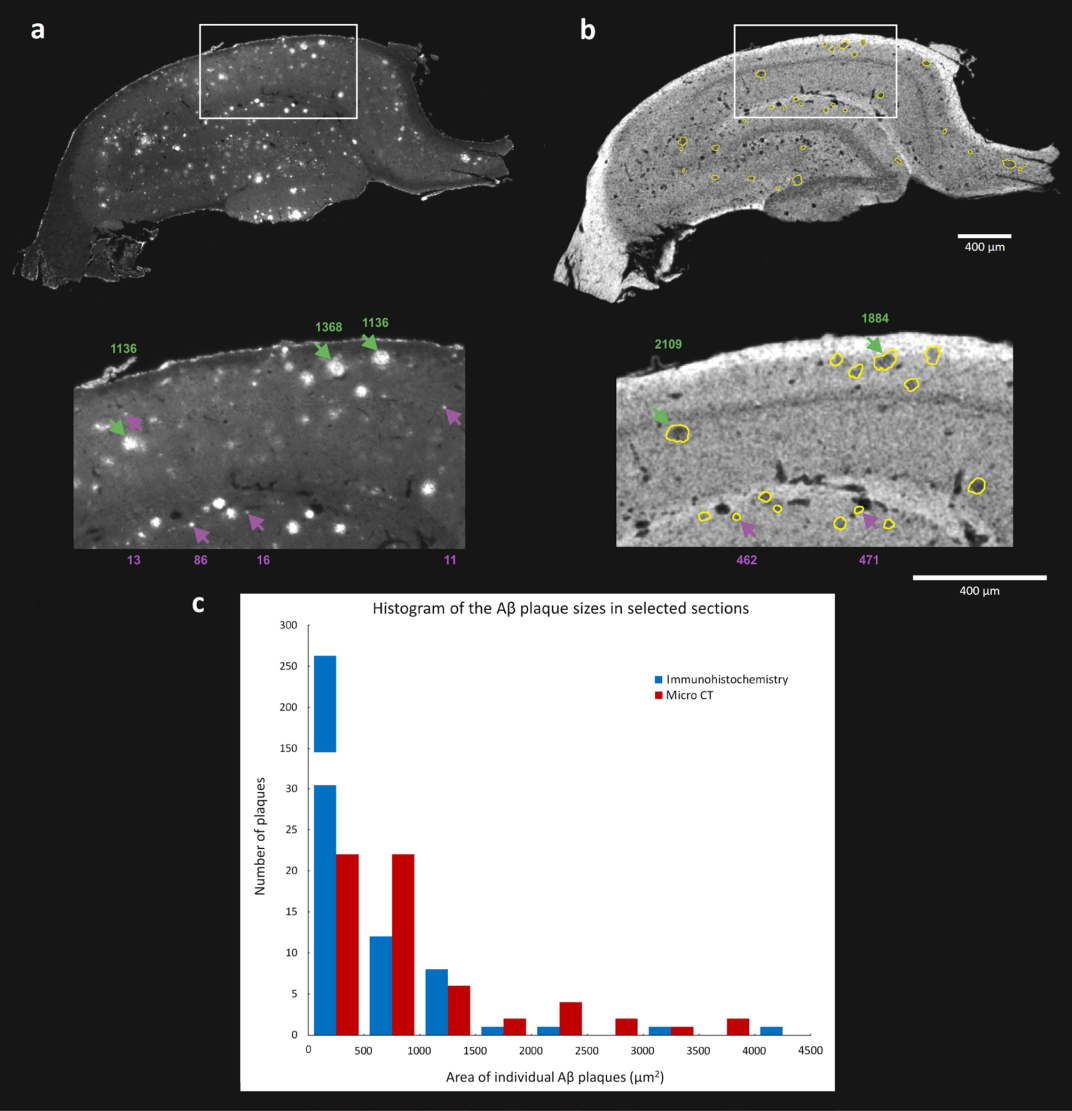
Comparison of the precision in identification of plaques in micro CT data versus the immunohistological detection: Comparison of corresponding sections from the same rat brain showing immunodetected Aβ plaques (**a**) and the micro CT data where the yellow line represents the manually selected plaque boundaries (**b**). The purple arrows indicate examples of the plaque cross sections corresponding to the smallest fraction (0–500 µm^2^) while the green arrows mark the larger size cases. The corresponding area values in µm^2^ are reported in proper colors above and under both pictures. The histogram (**c**) depicts the size interval distribution of detected Aβ plaque sections in the selected slices using immunohistochemistry and micro CT imaging, respectively^41–43^.

The next step was designed to assess the sensitivity of amyloid plaques detection using micro CT in contrast to the standard immunohistological staining. After the CT measurement, we sliced the isolated hippocampal tissue sample. Despite the dehydrated and iodine stained tissue turned out to be highly fragile while slicing, we were able to select four well preserved tissue sections and immunostained them for the Aβ presence. Then the corresponding micro CT sections were identified and their match was evaluated (Fig. 3a,b). The areas of Aβ deposit cross sections were marked by different observers. Each of the micro CT/histology image pairs was treated as independent. We obtained the plaque area median value of 597.9 µm^2^ (IQR 862.3 µm^2^) from CT data, whereas only 28.9 µm^2^ (IQR 105.4 µm^2^) from the histological sections, respectively. The plaque size histograms are depicted in Fig. 3c. This showed a considerable sensitivity difference between both methods, as the immunodetection returned more than one order higher amount of Aβ plaque sections in the lowest size category (0–500 µm^2^), accounting for its low median values. Besides the invisibility of smallest deposits on the scans, the comparison of the detailed images in Fig. 3a,b showed that the plaque border was harder to read in the micro CT data. This also might have caused an overestimation of the sizes of some of the smallest deposits. Consequently, they might have been marked as larger and so they were scored within the category of 501–1000 µm^2^.

**Figure 4:**
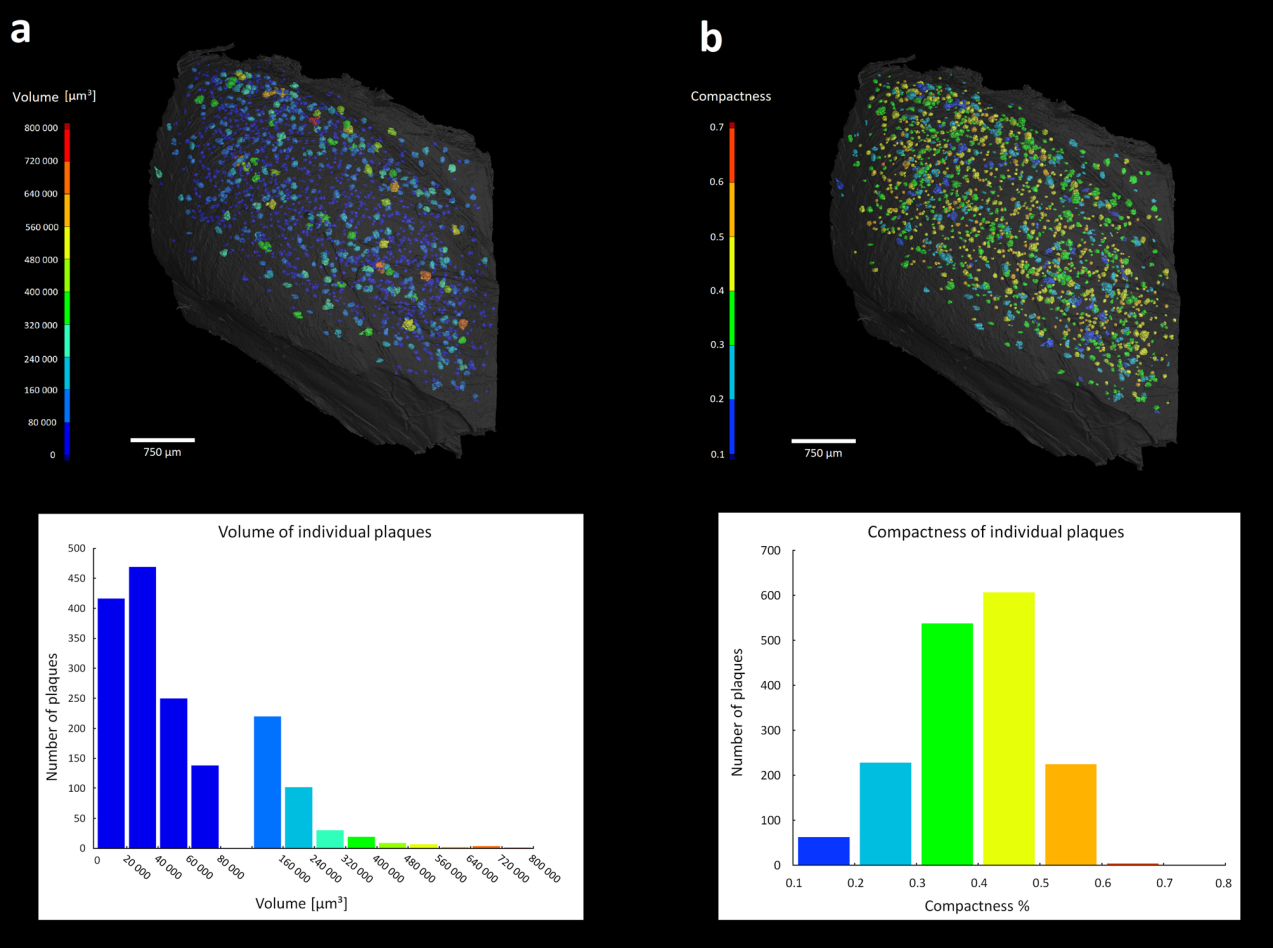
3D quantitative analysis of the Aβ plaques size and shape in hippocampus: Panel (**a**) shows the plaques 3D distribution color coded respective to their size, with the corresponding histogram in the same color scale on the right. Panel (**b**) illustrates the same plaques color scaled according their compactness (red codes for higher similarity to a sphere) with the respective histogram (right)^41,42^.

The data obtained from the micro CT scan of the dissected hippocampus was chosen for a subsequent 3D analysis. Since the Aβ plaques had similar contrast values as other tissue structures, they could not be detected with global thresholding methods. Hence, a manual segmentation of the plaques was performed. After defining the region of interest, all segmented plaques were counted and measured. In the dissected hippocampus (total volume = 13.88 mm^3^) we identified in total 1666 individual plaques. The volume of the smallest individual deposit was 895 µm^3^, indicating the lower limit for amyloid plaque identification in the present micro CT data. The biggest identified Aβ plaque had a volume of 721,552 µm^3^, the dataset of measured volumes was characterized by median value 38,423 µm^3^ and IQR of 57,512 µm^3^. The distribution of the Aβ plaques in 3D space with their color coded volumes is shown in Fig. 4a. Next, we assessed the shapes’ variability of Aβ plaques by measuring their compactness. The volume of the plaque was divided by the volume of the sphere circumscribed to the plaque. The values ranged between 0–1 where score of one represented a perfect sphere (Fig. 4b). The identified plaques had median of compactness at 0.396 with interquartile range 0.135. To alleviate supposed bias that could be caused by an eventual imperfection in defining boundaries in the smaller plaques, we additionally restricted the measurement to subset of plaques with volume larger than its median value (50% of the population). The corresponding compactness had a comparable median of 0.383 and IQR of 0.149.

**Figure 5:**
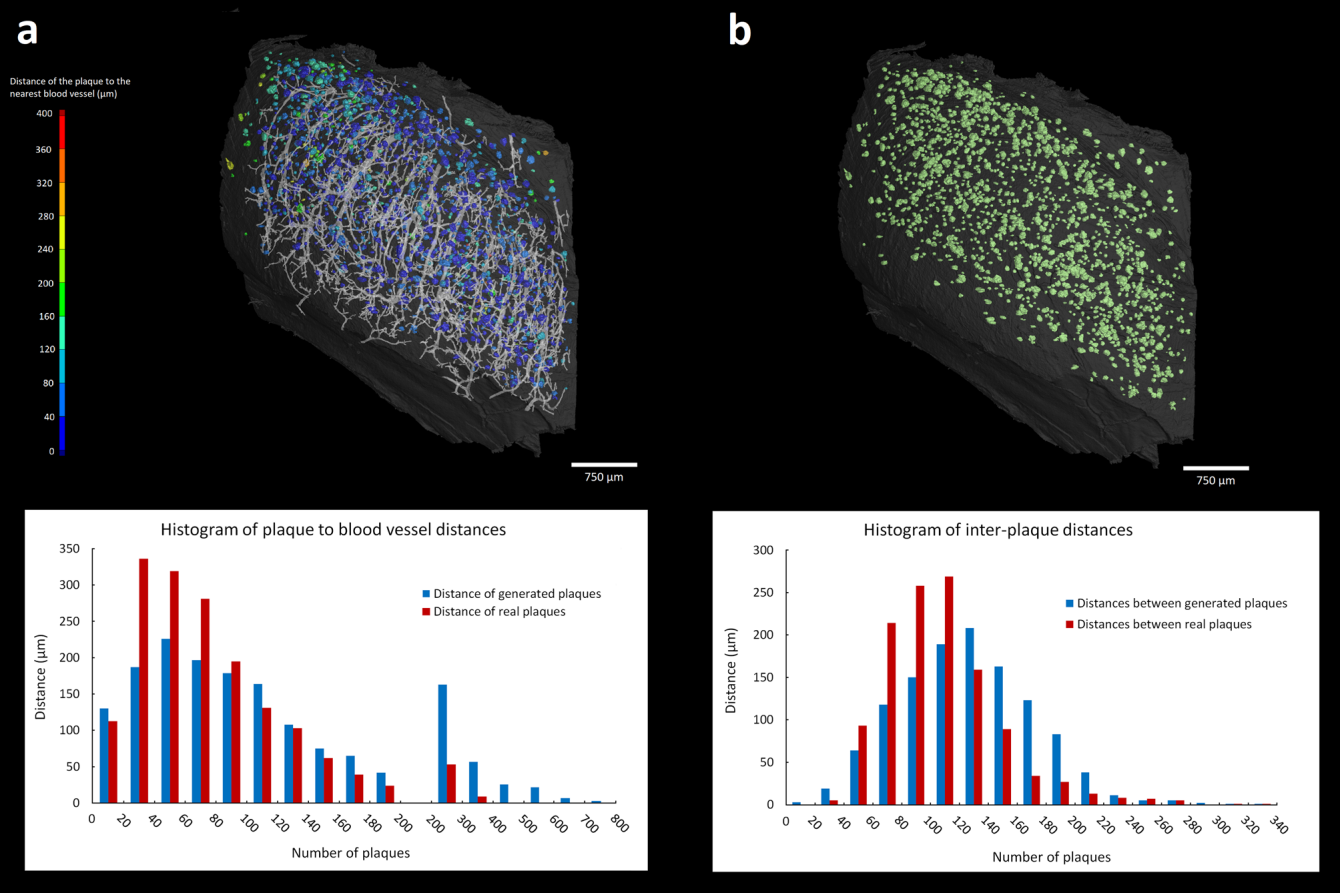
3D quantitative analysis of the plaques distribution in hippocampus: Horizontal view of the hippocampus with color coded distances of individual plaques to the nearest blood vessel (**a**) and the corresponding histogram showing the distances to the nearest blood vessel of randomly generated (in blue) and the real (in red) plaques, respectively. Horizontal view of the hippocampus depicting the distribution of the plaques inside the hippocampus (**b**) and the corresponding histogram showing the distribution of randomly generated plaques (in blue), and the distribution of real plaques (in red)^41,42^.

A precise 3D model of the plaque occurrence in the hippocampal sample allowed to quantify their spatial distribution including the relation to other labeled structures. We evaluated the distances of plaques to the nearest blood vessel and the inter-plaque distance (Fig. 5). The reconstructed 3D model returned the median distance between the plaques and the nearest vessel of 64.5 µm with IQR 62.3 µm (Fig. 5a). We then investigated whether their relation followed a non-random pattern. For each of the 1666 plaques we generated a random coordinate within the dissected part of hippocampus, leaving out the detected blood vessels. We measured the respective distances of generated “plaques” to a nearest vessel with median of 89.1 µm and IQR 103 µm. The comparison between the sets of experimental and randomly generated data returned the plaque-vessel distance significantly shorter in experimental data than in the random sample (Z = 10.95, p < 0.001, Mann Whitney U test), indicating the plaques tended to appear closer to the blood vessels than if their distribution was random. Finally we tested whether the plaques aggregated together irrespectively of the vessels. The same method of generating dataset with random positions was applied, and the distances between the two closest plaques were measured within the experimental and the random positions datasets, respectively (Fig. 5b). The median of distances from the tissue sample data was 101.1 µm with IQR = 46.5 µm, whereas the randomly generated dataset returned a distribution shift towards larger values (median distance 123.4 µm and IQR 62.7 µm). The Mann Whitney U test confirmed a statistically significant difference between the both measurements (Z = 11.97, p < 0.001).

## Discussion

In this paper, we present the absorption based contrast enhanced X-ray micro CT imaging performed on the industrial lab based CT system as an imaging tool for a 3D analysis of Aβ plaques of transgenic rat brains used as a model in Alzheimer disease research.

The detection of amyloid plaques in rodent brain by Computed Tomography (CT) has been attempted so far by using the mouse brain (*mus musculus*) and the experiments have been performed with synchrotron X-ray sources using variations of phase contrast imaging to visualize the Aβ plaques in a brain. TOMCAT beamline of the Swiss Light Source, Astolfo et al*.*^6^ demonstrated that synchrotron-based X-ray phase contrast imaging can be used for the 3D visualization and basic quantification of Aβ plaques in the mouse brain. At the same synchrotron facility the Pinzer et al*.*^7^ analyzed the neocortex of mouse brain via differential phase contrast imaging and accompanied their findings with complementary thioflavin S staining of the brain to confirm the presence of the Aβ plaques in brain in CT data. The diffraction enhanced imaging phase contrast X-ray imaging technique was applied by the Connor et al.^8^ at National Synchrotron Light Source, Brookhaven National Laboratory, USA. In that study, several scans of the one brain were needed in order to obtain a full scan of the brain. A comparison of the affected and wild type mouse brain was shown and the identity of Aβ plaques was confirmed by applying specific immunostaining on the Aβ plaques. The X-ray phase contrast tomography was applied for the purpose of the amyloid plaque imaging specifically to describe the microenvironment of the Aβ plaque at European Synchrotron Radiation Facility, Grenoble, France^9^. Finally, the research group of Noda-Saita et al.^10^ at High Energy Accelerator Research Organization, Ibaraki, Japan used phase-contrast X-ray CT imaging to describe the density of Aβ plaques supporting their findings with a complementary immunodetection of Aβ plaques in the mouse brain and with scans of control brains.

Unfortunately, the synchrotron facilities are not readily available for general use since the complicated imaging setups imply higher costs of equipment, its service and its maintenance. Moreover, the multiple scans have to be acquired to retrieve the information from the whole brain sample because of the sample’s size limitations. This restricts a wide use of synchrotron based CT imaging in the Alzheimer’s disease research, despite the obvious advantages for the quantification of amyloid plaques.

This fact led us to the idea of developing an imaging method for detection of amyloid plaques in the rat brain applicable to easily accessible industrial X-ray lab-based devices. Absorption based contrast enhanced X-ray micro CT imaging as an alternative to the synchrotron based phase contrast imaging techniques retrieves not only a 3D distribution of amyloid plaques with decent voxel resolution, but also provides valuable data for quantitative analysis of the amyloid plaques (total number, volume, shape).

The immunodetection of amyloid plaques performed on histological slices is the golden standard of identification of plaques in the brain tissue. In this paper, we used this method to validate our findings. In order to precisely correlate the micro CT data with the immunodetection of the plaques, we used the brain which had been previously scanned on micro CT. Then, we performed a classical histological sectioning and carried out the immunodetection of the plaques. The comparison of data from the same brain analyzed by both techniques showed a good agreement between immunodetection and micro CT imaging. Even though the immunodetection is indisputably more sensitive in detection of smaller plaques, it lacks a convenient approach to the 3D analysis across large brain volumes. This comparison thus highlights the advantages of contrast enhanced micro CT imaging. The immunostaining based affirmation of plaque identity detected by micro CT shows that the absorption-based contrast enhanced micro CT imaging is robust in detection of amyloid plaques. While its applicability as described in this report is not suitable for in vivo experiments for the staining protocol toxicity or because of the high irradiation dose, we believe the CT brain imaging approach in the context of Alzheimer’s disease research has a considerable potential for further development.

## Materials and methods

### Animals

Brains from three 18 months old female rats (two transgenic TgF-344 AD animals and one wild type control) obtained from the local breeding colony at Faculty of Medicine in Pilsen, Charles University, were used to collect the data. All protocols followed in this study were approved by the Ethical Committee of the Ministry of Education, Youth and Sports of the Czech Republic (approval no. MSMT-12048/2019-14) according to the Guide for the Care and Use of Laboratory Animals (Protection of Animals from Cruelty Law Act No. 246/92, Czech Republic).

### Micro CT staining

At the beginning of the experiments the rats were overdosed with pentobarbital and intracardially perfused with saline, followed by 4% PFA. Then the brains were manually extracted and post-fixed in 4% PFA for another 3 h. Afterwards, the samples were dehydrated in ethanol solutions of different concentrations which increased: 30%, 50%, 70%, 80% and 90%. The process lasted 12 h for each concentration. After the dehydration, samples were submerged in a staining solution consisting of 1% iodine in 90% methanol for 72 h, where the staining solution was refreshed after the first 24 h. Then the samples were washed in 50% ethanol and embedded in 1% agarose gel. In one of the transgenic animals, after the whole brain scan, the dorsal hippocampus was dissected and embedded in 1% agarose gel for another scan.

### Micro CT measurement

Before scanning, all samples were embedded in 1% agarose gel in 15 ml Falcon tubes in order to prevent movement during the imaging procedure. The micro CT scanning was performed using a laboratory system GE Phoenix v|tome|x L 240 (GE Sensing & Inspection Technologies GmbH, Germany), equipped with a 180 kV/15 W maximum power nanofocus X-ray tube and a high contrast flat panel detector dynamic 41|100 (number of pixels: 4048 × 4048 px, pixel size 100 μm). The measurements were carried out in an air-conditioned cabinet (21 °C). The parameters for each scan are indicated in the Table 1. The tomographic reconstruction was realized by software GE phoenix datos|× 2.0 (GE Sensing & Inspection Technologies GmbH).

**Table 1:** Micro CT scan settings.

Sample	Rat brain F60 +	Rat brain F61-	Rat brain F87 +	Hippocampus F87 +
Voltage [kV]	60	60	60	60
Current [µA]	200	200	200	200
Timing [ms]	600	600	600	700
Source spot size [µm]	5	5	5	5
Sample/source distance [mm]	40.8	40.8	40.8	11.9
Sample/detector distance [mm]	864.9	864.9	864.9	785.4
Images	2200	2200	2200	2400
Time [min.]	80	80	80	100
Voxel size [µm^3^]	9	9	9	3

### Micro CT data processing

All 3D visualizations and measurements were performed in VG Studio MAX 3.4 software (Volume Graphics GmbH)^41^. The segmentation of plaques in the hippocampus sample was carried out using Avizo 9.5 software (Thermo Fisher Scientific)^42^. In the first step we isolated the hippocampus from the background by creating the corresponding region of interest (ROI). Blood vessels were segmented by global thresholding based on the grey level (vessels appeared as the darkest part since they did not contain any iodine), continuity, and their resulting prolonged and branched 3D shape. The amyoid plaques were segmented manually in Avizo 9.5 software by selecting the plaque areas across the sections under the following criteria: the area of the plaque should be distinguishably darker then the surrounding tissue and its volume should extend across at least 3 following sections. The boundaries of smaller plaques were selected section by section, in bigger plaques (some were spread over more than 30 sections) we interpolated between every other slice while any inconsistencies were additionally manually corrected.

For the analysis of Aβ distribution we generated datasets of plaques coordinates (Matlab R2020a). First, the coordinates of plaques centroids were extracted from the segmented 3D binary data to represent the experimental dataset. Then the random dataset of “plaque’s” centroids was created within the volume of the sample leaving out its vascular system. The resulting coordinates then represented centroids of randomly distributed Aβ plaques with total amount corresponding to the experimental dataset. The analysis of Aβ plaque position in relation to the blood vessels was carried out in VG studio. Both real plaques centroid positions and simulated plaques centroid positions were imported in form of binary image data. The distance of the centroid to the nearest blood vessel was measured via pore analysis module. Subsequently, the intra-plaques distances were analyzed separately in simulated plaque centroids and real plaque centroids. The nearest neighboring centroid of each centroid was found using 3D Euclidean distances analysis in Matlab R2020a. Repeating combinations of the nearest neighbors were excluded from the subsequent analysis.

### Statistical analysis

The non-parametric Mann–Whitney test was applied to compare the detected and random datasets (https://www.statskingdom.com/170median_mann_whitney.html).

### Histology and immunohistochemical labeling

After the CT measurements, the sample tissue was embedded in a paraffin block and cut into 10µ thick sections using an Automated Microtome (Leica RM2255). The slices were then deparaffinized and rehydrated. Phosphate buffer saline (PBS, 0.1 M) was applied for washing. To block the non-specific binding, normal goat serum (ab138478, Abcam) was used. The sections were then incubated overnight at 4 °C with the primary antibody (ab2539, Abcam, 1:200, 1 mg/ml) against amyloid beta. Next day, the sections were washed with 1X PBS (0.01 M) thrice (5′, 10′ and 15′) to remove the unbound antibody remnants. Staining was visualized with an Alexa Fluor 647-conjugated goat anti-rabbit antibody (Jackson ImmunoResearch Laboratories) applied in 1:500 dilution at 37 °C for 4 h. All the sections were then counterstained with DAPI to label the nuclei and glass mounted using fluoroshield mounting medium (Merck). The Amyloid beta plaques were visualized using fluorescent microscopy (Olympus), and quantified in open source Fiji image analysis software^43^.

## Supplementary Information

Supplementary Information

Supplementary Video 1.

## Data Availability

Datasets used in this publication are available on request at corresponding authors.
