# Supplementary material for: Contrast enhanced X-ray computed tomography imaging of amyloid plaques in Alzheimer disease rat model on lab based micro CT system
Source: Sci Rep. 2021 Mar 16;11:5999. doi: 10.1038/s41598-021-84579-x (PMC7966753; doi:10.1038/s41598-021-84579-x)
Supplement: Supplementary file 1 — Supplementary Information [file 41598_2021_84579_MOESM1_ESM.docx]

**Contrast enhanced X-ray Computed Tomography imaging of amyloid plaques in Alzheimer disease rat model on lab based micro CT system**

Michaela Kavkova^1^**^*^**, Tomas Zikmund^1^, Annu Kala^2^, Jakub Salplachta^1^, Stephanie L. Proskauer Pena^2^, Josef Kaiser^1^, Karel Jezek^2^**^*^**

**Supplementary Information:**

**
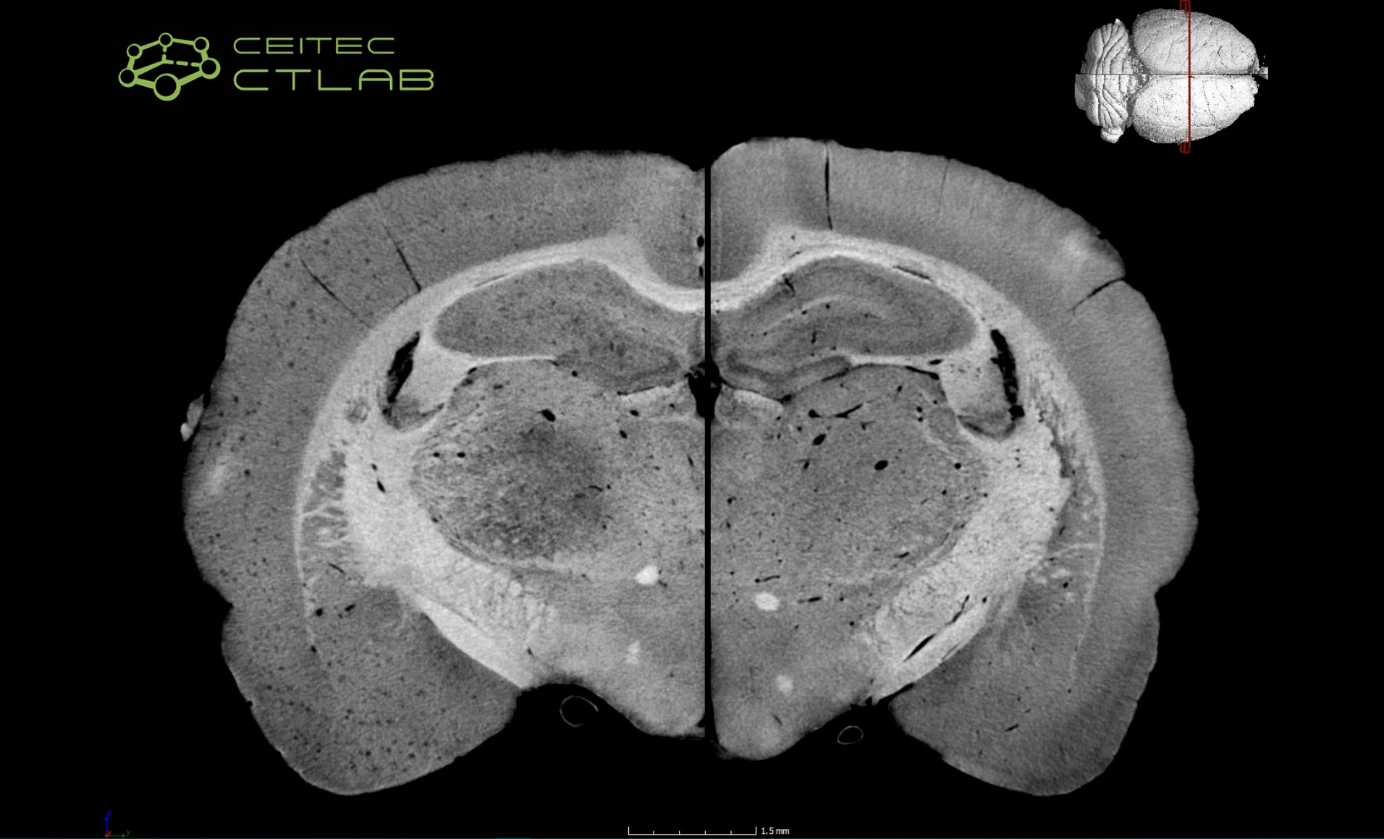
**

Video 1: Comparison of the transgenic TgF-344 AD brain (left side) and wild type control (right side) in micro CT sections throughout the entire brain

https://www.vutbr.cz/www_base/vutdisk.php?i=249656aba8
